# Description of morphological characteristics of wheat spike as a digital certificate in the SpikeDroidDB database

**DOI:** 10.18699/vjgb-26-37

**Published:** 2026-04

**Authors:** E.G. Komyshev, Yu.V. Kruchinina, V.S. Koval, A.A. Poteshkina, N.V. Petrash, V.V. Piskarev, N.P. Goncharov, D.A. Afonnikov

**Affiliations:** Institute of Cytology and Genetics of the Siberian Branch of the Russian Academy of Sciences, Novosibirsk, Russia Kurchatov Genomic Center of ICG SB RAS, Novosibirsk, Russia; Institute of Cytology and Genetics of the Siberian Branch of the Russian Academy of Sciences, Novosibirsk, Russia Kurchatov Genomic Center of ICG SB RAS, Novosibirsk, Russia; Institute of Cytology and Genetics of the Siberian Branch of the Russian Academy of Sciences, Novosibirsk, Russia Kurchatov Genomic Center of ICG SB RAS, Novosibirsk, Russia; Institute of Cytology and Genetics of the Siberian Branch of the Russian Academy of Sciences, Novosibirsk, Russia Siberian Research Institute of Plant Production and Breeding – Branch of the Institute of Cytology and Genetics of the Siberian Branch; Institute of Cytology and Genetics of the Siberian Branch of the Russian Academy of Sciences, Novosibirsk, Russia Siberian Research Institute of Plant Production and Breeding – Branch of the Institute of Cytology and Genetics of the Siberian Branch; Institute of Cytology and Genetics of the Siberian Branch of the Russian Academy of Sciences, Novosibirsk, Russia Siberian Research Institute of Plant Production and Breeding – Branch of the Institute of Cytology and Genetics of the Siberian Branch; Institute of Cytology and Genetics of the Siberian Branch of the Russian Academy of Sciences, Novosibirsk, Russia; Institute of Cytology and Genetics of the Siberian Branch of the Russian Academy of Sciences, Novosibirsk, Russia Kurchatov Genomic Center of ICG SB RAS, Novosibirsk, Russia

**Keywords:** wheat, spike, morphometry, digital phenotyping, database, collection, пшеница, колос, морфометрия, цифровое фенотипирование, база данных, коллекция

## Abstract

It has been repeatedly shown that spike productivity is the main component of wheat yield. The main spike parameters related to productivity are size, the number of grains and spikelets per spike, and the presence or absence of awns. In modern genetic research, morphometric analysis of hundreds and thousands of spikes is required to determine the loci that control spike productivity traits. On the other hand, thousands of accessions in modern collections of wheat genetic resources need detailed description. These considerations motivate the development of digital technologies for describing spike traits in wheat, which can be achieved through image analysis methods. These methods allow for automated acquisition of trait values that can serve as the basis for digital plant collections. Here we propose an extended set of spike characteristics obtained both manually and through digital image analysis and present plant characterization. These data form the basis of the updated version of the SpikeDroidDB database (http://spikedroid.biores.cytogen.ru/). The digital description of the spike consists of two blocks. The block of uploaded data includes a description of the plant and contains five tables: collection; variety sample (year of cultivation (vegetation), sowing identifier, taxonomic information, etc.), planting site, and characteristics of the spike determined manually (length, width of frontal and lateral views, type and color of the spike, etc.) The block of extracted features includes spike characteristics obtained by digital phenotyping and contains six tables: characteristics of the spike outline in the image; characteristics of the quadrangle model, values of the color components of the spike, dominant colors of the spike, and texture characteristics of the spike in the image. The most illustrative and significant features of the spike have been identified, allowing for the formation of the spike digital certificate, which includes size, shape, and color features derived from the digital images. The features forming the digital certificate have been compared between two wheat species, T. aethiopicum and T. carthlicum. It is shown that the features of the digital certificate allow for a clear representation of the spike model and the identification of distinct parameters: colors of the spike and awns and roundness of the frontal view of the spike. The database interface has been supplemented with the ability to upload data on plant and spike characteristics, as well as their images, in the batch mode.

## Abstract (ru)

При анализе структуры урожая пшеницы неоднократно показано, что его выраженность зависит от продуктивности колоса. Основные характеристики колоса, которые связаны с продуктивностью, это прежде всего масса 1000 зерен, число зерен и колосков в колосе, его размеры, наличие/отсутствие остей и другие. В современных генетических исследованиях для идентификации локусов, контролирующих признаки продуктивности колоса, требуется морфометрия сотен и тысяч колосьев. С другой стороны, современные коллекции генетических ресурсов содержат тысячи образцов, которые также требуют своего детального описания. Все это обусловливает необходимость развития цифровых технологий описания признака колоса пшеницы, которые могут быть достигнуты на основе методов анализа изображений. Эти методы позволяют автоматически получать значения набора признаков, которые могут служить основой формирования цифровых коллекций растений. В настоящей работе предложено цифровое описание колоса пшеницы на основе характеристик, полученных как вручную, так и на основе анализа цифровых изображений, а также характеристик растений, у которых был взят колос. Эти данные положены в основу обновленной версии базы данных SpikeDroidDB (http://spikedroid.biores.cytogen.ru/). Цифровое описание колоса состоит из двух блоков. Блок загружаемых данных содержит описание растения и включает 5 таблиц: коллекция, сортообразец (год выращивания (вегетация), посевной номер, таксономическую информацию и др.), место выращивания, характеристики колоса растений, оцененные экспертом вручную (длину, ширину фронтальной и боковой проекций, тип и цвет колоса и др.). Блок извлекаемых характеристик включает признаки колоса, полученные в результате цифрового фенотипирования, и состоит из шести таблиц: характеристики контура колоса на изображении; характеристики модели четырехугольников, компоненты цвета колоса, доминантные цвета колоса, текстурные характеристики колоса на изображении. Было проведено выделение наиболее наглядных и информативных характеристик колоса, которые позволили сформировать цифровой паспорт колоса, включающий признаки размера, формы и цвета, определенные на основе анализа цифровых изображений. Проведено сравнение признаков, формирующих цифровой паспорт, у двух видов пшеницы, T. aethiopicum и T. carthlicum. Показано, что признаки цифрового паспорта позволяют наглядно представить модель колоса, а также выявить достоверно различающиеся параметры (цвет колоса и остей, округлость фронтальной проекции колоса). В интерфейс базы данных также добавлена возможность пакетной загрузки данных о характеристиках растения и колоса, а также их изображений.

## Introduction

It has been repeatedly shown that spike productivity is the main component of wheat yield (Moiseeva, 2017; Romanov, Pimonov, 2018; Demina, 2022; Shuklina et al., 2022). The most significant characteristics for yield are spike size, the number of spikelets and grains, and degree of compactness. For wheat processing, it is also important to consider the presence or absence of awns, spike color, and so on. The investigation of genes that control these traits is of particular interest to geneticists and breeders (Guo et al., 2018; Garland-Campbell, 2022). In such studies, it is crucial to evaluate the diversity of spike traits across a large number of accessions, which allows for the identification of associations between varieties in spike characteristics and nucleotide substitutions in the wheat genome (Wu et al., 2012; Xu et al., 2024).

The study of wheat and its relatives also relies on the identification of various spike traits, particularly those that allow for plant taxonomy, biological material classification (Spagnoletti Zeuli, Qualset, 1987; Khanjari et al., 2008; Goncharov, 2009), and the evaluation of breeding trials (Methodology..., 2019). Such traits include spike shape (Konopatskaia et al., 2016), pubescence (Goncharov et al., 2007), the presence or absence of awns (Smolenskaya et al., 2022), spike and awn color (Lyapunova, 2017; Smolenskaya et al., 2022), and compactness (Vavilova et al., 2017). These morphological traits are used by geneticists and breeders in analyzing wheat collections and breeding programs, but their assessment is labor-consuming due to the large volume of material analyzed, often involving hundreds or thousands of specimens (Pakul, Sherina, 2009; Piskarev et al., 2018; Zuev et al., 2019).

To enhance the efficiency of trait determination in modern genetics and breeding, phenotyping technologies based on digital image analysis are employed. These technologies automate the process of phenotypic characteristic assessment, thereby reducing labor costs, enabling evaluations of thousands of samples (Afonnikov et al., 2016; Meraj et al., 2024), and permitting the application of machine learning methods (Murphy et al., 2024). For wheat spikes, image-based phenotyping methods have also been intensely developed recently. They primarily allow for the determination of morphological characteristics of the spike, such as length, width, and density (Genaev et al., 2019). X-ray imaging enables detailed three-dimensional descriptions of spike structures down to individual grains (Ling et al., 2023). Machine learning methods can automatically determine the number (Genaev et al., 2026) and shape (Niu et al., 2024) of spikelets, as well as assess the presence or absence of pubescence (glume hairs) (Artemenko et al., 2024).

Digital phenotyping allows for the determination of a significantly larger number of plant characteristics, much more than a breeder could previously examine (Afonnikov et al., 2016). Some of these characteristics, such as spike length or the number of grains per spike, are directly related to yield (Moiseeva, 2017; Romanov and Pimonov, 2018; Demina, 2022; Shuklina et al., 2022). Others are indirectly related to productivity, e. g., grain weight, which can be estimated from the area of its view on an image. Some digital characteristics are difficult to interpret agrobiologically, but they can be effectively used for spike classification employing machine learning methods (Bi et al., 2010). The choice of digital traits is crucial for creating digital collections–databases that describe plant phenotypes (Conejo-Rodríguez et al., 2024). On the one hand, these traits should be familiar to breeders working with the databases (Methodology…, 2019), whereas on the other, they should be sufficiently representative to solve classification tasks.

Previously, we developed the SpikeDroidDB database, which stores information about wheat spike samples, including expert-assessed trait descriptions (Genaev et al., 2018). Here we provide an expansion and systematization of the set of digital features for describing spikes in this database, such as quadrangle model parameters (Genaev et al., 2019) and spike and awn color characteristics. The database interface has been supplemented with the ability to upload information, including expert-assessed sample characteristics and spike images, in the batch mode. As a result, the number of spike sample descriptions in the database exceeded 1700.

## Materials and methods

**Plant material. **The database was supplemented with descriptions of spike samples from the Siberian Gene Pool bread wheat variety collection of the Siberian Research Institute of Plant Production and Breeding and the Institute of Cytology and Genetics, SB RAS (Piskarev et al., 2018). Plants were grown in 2019–2020 in experimental fields of the Siberian Research Institute of Plant Production and Breeding – Branch of the Institute of Cytology and Genetics of the Siberian Branch of the Russian Academy of Sciences (Krasnoobsk, Novosibirsk region)

**Fig. 1.**
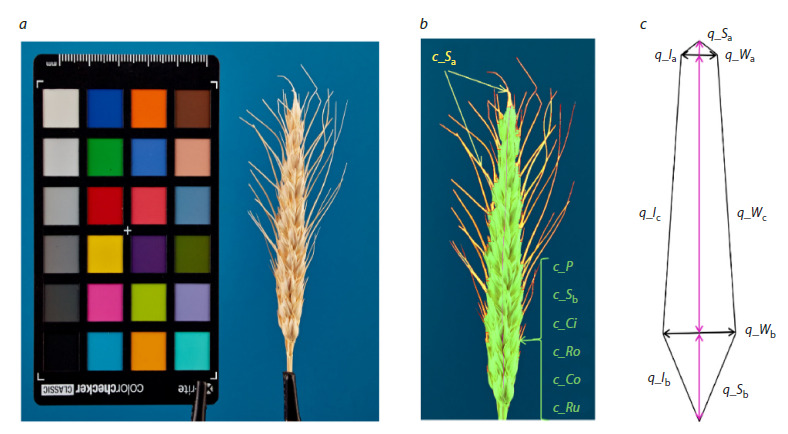
Morphometric characteristics of the spike. (a) Image of the spike placed vertically on a clip, frontal view. (b) Results of image segmentation showing the spike body (light green), awns (yellow), and background (blue). The spike axis is indicated by a pink line. (c) Quadrangle model for the spike. Feature designations in (b) and (c) follow Table S1.

**Digital phenotyping.** To obtain spike images, we used the ‘on a clip’ protocol described previously (Genaev et al., 2018, 2019). The spike was placed vertically on a clip against a backdrop of blue paper. Images were taken using a Canon 350D digital camera with an EF-S 18-55mm f/3.5-5.6 lens. X-Rite Mini ColorChecker Classic color chart was positioned within the frame for scale and color calibration (Fig. 1а). The spikes were photographed in four views: frontal (the widest view), back, and two side views. To obtain digital characteristics of the spike, the images were segmented into background, color chart, spike body, and awns using deep machine learning methods (Artemenko et al., 2024). The segmented image was then processed with the WERecognizer program (Genaev et al., 2019) to estimate morphometric and color characteristics of the spike. The morphological characteristics included a set of features for a quadrangle model symmetrized relative to the spike main axis (Komyshev et al., 2024), Figure 1b, c; a set of outline features of the spike body in the image (length, perimeter, area, circularity, roundness, solidity, and rugosity); and the area of the awns. In total, 19 morphological features of the spike and awns were analyzed.

For the spike and its awns, digital color characteristics were determined. The method is based on a previously applied approach for analyzing wheat grains (Afonnikov et al., 2022). For all pixels of the spike body, the average values of color components were calculated in four color spaces: RGB, Lab, HSV, and YCrCb (Komyshev et al., 2020), totaling 12 characteristics. Additionally, three dominant colors and their corresponding component values were identified. Dominant colors are defined as centroids of clusters in the RGB component space obtained by the k-means method, assuming k = 3 (Afonnikov et al., 2022). These colors characterize the three largest groups of pixels similar in color in the spike image and satisfactorily describe its color heterogeneity. Typically, the first cluster (dominant color) has component values close to the average values across the entire spike image. Color characteristics for awn pixels were determined in a similar way.

Additionally, texture features of the spike area in the image were calculated. These features characterize the structural heterogeneity of the object in the image, which may arise due to uneven coloring or the surface structure of the glumes (e. g., pubescence). A distinguishing feature of simple texture is regularity, with repetitive or partially reproducible elements on a certain surface or object. Two types of structural features were calculated: those based on the Grey Level Co-occurrence Matrix (GLCM, 10 characteristics) and the Gray Level Runlength Matrix (GLRM, 6 characteristics) (Komyshev et al., 2020).

**Fig. 2.**
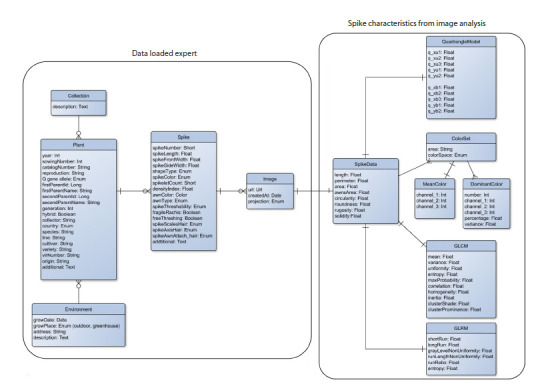
Logical structure of the updated version of the SpikeDroidDB database. On the right is the user-input data block. On the left is the data block obtained through digital phenotyping of images.

**SpikeDroid database structure. **The aforementioned spike characteristics obtained through the analysis of digital images, together with the characteristics determined by expert evidence and plant parameters, are stored in the updated version of the SpikeDroidDB database, whose logical structure is presented in Figure 2.

The structure of the updated version of the SpikeDroidDB database includes two data blocks. The block of data uploaded by experts contains five tables and describes the collection and the plant whose spike is analyzed. The Collection table contains a text field for the name of the collection and a description field. The Plant table includes 20 fields: year of cultivation (vegetation), accession number in the collection, sowing identifier, reproduction number, Q-gene allele (Vavilova et al., 2020), information on parent plants, taxonomic information, and origin of the variety/line. The Environment table contains information about the time and location of cultivation, including the address of the originating institution. The Spike table contains characteristics determined manually: spike number on the plant, spike length, frontal and lateral view widths, spike type, color, number of grains, awn color and type, pubescence characteristics of the glume, spike threshability, rachis fragility, and awn attachment type. Additionally, this block contains information about the spike image (Image table), in cluding the view number, date of acquisition, and a link to the image file.

The block of extracted features includes spike characteristics obtained by digital phenotyping (see above). It includes seven tables. The SpikeData table includes characteristics of the spike outline in the image. The QuadrangleModel table presents characteristics of the quadrangle model. The Mean- Color table stores mean color characteristics of the spike. The DominantColor table contains dominant color characteristics of the spike. The GLCMTexture table contains texture parameters for GLCM. The GLRMTexture table contains texture parameters for GLRM.

Digital spike certificate. Among the numerous shape and color characteristics of a spike presented in the SpikeDroidDB database (see above), several key features should be highlighted that can be used for a reliable assessment of similarities/ differences in spike shape, size, and color. These characteristics can also be used for visually demonstrating the similarities or dissimilarities between spikes of different accessions from the plant collection. Previously, we demonstrated that simplified geometric model features of the spike based on quadrangles and outline characteristics of the spike from images can be used to identify both interspecies and intraspecies differences in spikes (Komyshev et al., 2024). These features allow for high-precision species identification of plants. We obtained grounds for forming the digital spike certificate by choosing those features of shape and size obtained from the analysis of digital images that were most visually informative and sufficient for classification tasks. These features were supplemented with color characteristics of the spike and awns. The list of features forming the digital certificate is provided in Table S1 of the Supplementary File1. For each spike image, it includes: 11 parameters of the quadrangle model (Komyshev et al., 2024), 7 outline features of the spike (Genaev et al., 2019), 3 color parameters of the spike (average R, G, B components of the spike body pixels), and 3 color parameters of the awns (average R, G, B components of the awn pixels).

Supplementary Materials are available in the online version of the paper: https://vavilovj-icg.ru/download/pict-2026-30/appx19.pdf


For most spikes in the database, at least four views are stored. Therefore, the digital certificate of each spike includes averaged characteristics for the frontal/back and two side views. In cases where only one view is available for a spike image, usually the frontal view following the ‘on the table’ protocol (Genaev et al., 2018), the certificate contains only the features derived from that single image.

**Database implementation.** The database is implemented using the Drupal content management system (https://www. drupal.org). Data storage is provided by a relational database managed by the MySQL database system, which is deployed on the server of the “Bioinformatics” shared access computational center running CentOS Linux.

## Results

**Fig. 3.**
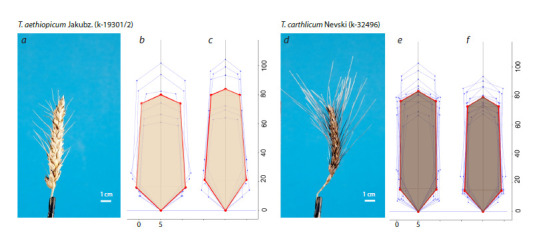
Graphical representation of the spike digital certificate parameters for two wheat species: T. aethiopicum Jacubz. (a–c) and T. carthlicum Nevski (d–f). Digital images of the spike are shown in panels a and d; models of the spike for the frontal view are shown in panels b and e; models of the spike for side views are shown in panels c and f. Thin blue lines represent the geometric models of the spike for individual plants, while thick red lines show polygons representing models for the mean values of spike parameters. The fill color of the red polygons corresponds to the averaged color of the spike outline pixels. The length scales in mm along the X- and Y-axes for the spike models differ by a factor of 5 for better visualization of the spike shape characteristics.

Two examples of spike visualization based on the characteristics presented in their digital certificates are shown in Figure 3. They represent two wheat species from N.P. Goncharov’s collection: Triticum aethiopicum Jacubz. (VIR accession number k-19301/2, represented by 6 plants) and T. carthlicum Nevski (VIR accession number k-32496, 12 plants).

The figure demonstrates the similarity in spike length between the two wheat species. However, their shapes differ slightly. The frontal view of T. aethiopicum appears more rounded, whereas that of T. carthlicum is more elongated. Differences in the size and number of awns are clearly visible in the images: T. aethiopicum has fewer and shorter awns compared to T. carthlicum. Additionally, the colors of the spikes and awns differ: They are lighter in T. aethiopicum and darker in T. carthlicum.

We conducted a statistical test to confirm the differences between the mean values of the digital certificate parameters for the two species. Significant differences were found in five shape features (all related to outline characteristics but not the model parameters) and all color characteristics (both for the spike and awns) in both the frontal and side views. The results are provided in the supplementary material (Table S2). They are in good agreement with the visual assessment (Figure 3). For the frontal view, significant differences were observed in the following indices: area of awns (c_Sa), circularity (c_Ci), roundness (c_Ro), and integrity (c_So). For the side view, significant differences were observed only in the area of awns. Significant differences were noted in all color components of the spike and awns in both the frontal and side views.

Thus, the features forming the digital certificate of the spike allow for a clear representation of similarities and differences in spike characteristics between the two wheat species, as well as the recognition of statistically significant characteristics.

**Fig. 4.**
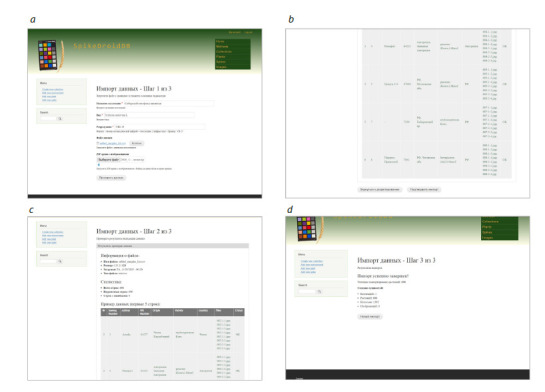
Visualization of the user interface during the main steps of batch uploading data about spikes and their images into the SpikeDroidDB database. (a) Input of primary dataset characteristics (wheat species, reproduction number, field for uploading the collection description file). (b) Visualization of the list of uploaded data. (c) Visualization of information about the uploaded data. (d) Completion of data import.

The possibility to upload data for multiple spikes at once simplifies the user’s interaction with the database. The user interface for importing data in this case is shown in Figure 4. It demonstrates the step-by-step implementation of uploading multiple images and descriptions of spikes.

**Fig. 5.**
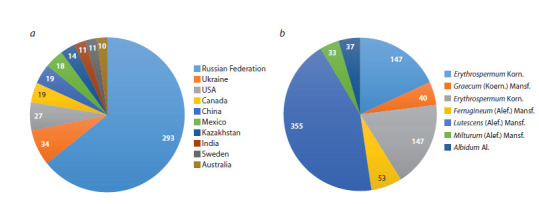
Statistics of bread wheat samples (reproduction 2019) in the SpikeDroidDB database. Distribution of samples: (a) by countries of origin, (b) by varieties

As an example, Figure 5 provides the statistics of the uploaded data for samples from the ‘Siberian Wheat Collection’ grown in 2019. A total of 696 samples were grown and analyzed. Their distributions by countries of origin, i. e., the country from which each specific variety sample originated (the top 10 most represented in the analyzed collection), and by varieties (most represented in the analyzed material) are shown.

## Discussion

The development of modern phenotyping methods allows for the digitization of spike characteristics for subsequent computer analysis. Previously we demonstrated that digital characteristics of spike shape and size correlate with equivalent biological characteristics measured manually (Komyshev et al., 2024). The same work showed that the combined use of digital and classical characteristics allows for high-precision classification of plants into species. Furthermore, such representation enables the identification of traits that significantly vary among specimens of the same species (Komyshev et al., 2024). All of this underscores the usefulness of both classical and digital spike characteristics for geneticists and breeders in describing spike traits. We applied these considerations to the development of the digital spike certificate, which includes descriptions of plant characteristics, their growing site locations, spike traits assessed by experts, and digital characteristics derived from image analysis. This comprehensive representation allows for a closer description of spike properties. Digital characteristics of the spike include quadrangle model parameters, outline properties of the spike in images, and a set of color and texture parameters. Thus, digital phenotype parameters, in conjunction with classical trait sets, make plant descriptions more detailed and help in classification (Conejo- Rodríguez et al., 2024).

The digital representation of phenotypic characteristics of plants is a crucial step in the development of modern plant genetic resource collections (Thormann et al., 2012; Heberling, 2022). On the one hand, it enables rapid assessment of the vast diversity of phenotypic traits in collections. On the other hand, digital data from collections can be more effectively utilized in breeding programs (Dipta et al., 2023). This highlights the advantage of describing the properties of germplasm banks and breeding collections in a digital form for the purpose of genetic research and breeding. Further development of phenotyping methods and the selection of the most important digital characteristics will enable a deeper understanding of the manifestation of phenotypic traits, thereby potentially enhancing the efficiency of identifying genes that control them.

## Conclusion

A comprehensive system for the digital description of wheat spike was developed. It integrated both traditional manual evaluation methods and modern digital phenotyping technologies. As a result, the structure of the SpikeDroidDB database was updated for the comprehensive representation of spike properties. The updated structure includes two main data blocks. The block of uploaded data contains fundamental information about the collection and specific variety sample characteristics, presented in five interrelated tables. Emphasis was placed on agronomic and taxonomic information, growing conditions, and basic spike characteristics determined manually by experts. The block of extracted features comprises an extended set of parameters obtained through digital image analysis. It includes six categories of data: outline characteristics of the spike, quadrangle model parameters, color characteristics (including dominant colors), and texture features. An important functional enhancement was the implementation of batch data upload capability, significantly simplifying the process of entering information about plants and uploading their images, as well as their subsequent statistical analysis.

The most visually informative and relevant characteristics of a spike were identified, allowing the formation of a digital certificate of the spike, which includes size, shape, and color features determined based on digital image analysis. We compared the features forming the digital certificate between two wheat species, T. aethiopicum and T. carthlicum, and demonstrated that the digital certificate features allow for a clear representation of the spike model and the identification of significantly differing parameters (color of the spike and awns and roundness of the frontal view of the spike). Thus, the efficiency of describing spike characteristics for research in breeding and genetics is enhanced, enabling a comprehensive analysis of plant morphological features

## Conflict of interest

The authors declare no conflict of interest.
